# Regular Testing of HIV and Sexually Transmitted Infections With Self-Collected Samples From Multiple Anatomic Sites to Monitor Sexual Health in Men Who Have Sex With Men: Longitudinal Study

**DOI:** 10.2196/40996

**Published:** 2022-11-18

**Authors:** Ngai Sze Wong, Tsz Ho Kwan, Denise P C Chan, Grace C Y Lui, Shui Shan Lee

**Affiliations:** 1 Jockey Club School of Public Health and Primary Care The Chinese University of Hong Kong Hong Kong China; 2 Stanley Ho Centre for Emerging Infectious Diseases The Chinese University of Hong Kong Hong Kong China; 3 Department of Medicine & Therapeutics The Chinese University of Hong Kong Hong Kong China

**Keywords:** HIV testing, STI testing, self-sampling, internet-based testing, men who have sex with men, HIV, monitoring, sex, infection, prevention, community, engagement, cohort study, testing

## Abstract

**Background:**

Regular HIV and sexually transmitted infection (STI) testing for men who have sex with men (MSM) is an important means of infection prevention, the adoption of which remains suboptimal in the community.

**Objective:**

On the hypothesis that engagement plays an important role in sexual health monitoring, this study aimed to pilot-test internet-based HIV and STI testing with self-sampling to enhance engagement of MSM with regular testing.

**Methods:**

This 1-year cohort study was conducted on HIV-negative MSM aged 18 years or older. A designated website was set up to enable participants to make appointments for baseline and follow-up visits at 3-monthly intervals. On-site blood sampling was performed for HIV and syphilis tests, along with self-collection of pharyngeal swabs, rectal swabs, and urine samples for *Chlamydia trachomatis* (CT) and *Neisseria gonorrhoeae* (NG) testing. Full engagement, as defined by having made at least 3 visits over a 6-12 months’ follow-up period, was compared with partial engagement in the bivariable logistic regression model.

**Results:**

Between August 2019 and October 2020, 204 MSM were recruited, after the exclusion of 2 baseline HIV-positive MSM. The majority (189/204, 92.7%) were Chinese, the median age was 31 (IQR 26-39) years, and 58.0% (116/200) had experience with pre-exposure prophylaxis (PrEP) at baseline. Full engagement (146/204, 71.6%) was associated with incident STI during the follow-ups (odds ratio [OR] 4.23, 95% CI 1.63-10.94), seeking a medical referral after STI detection (OR 10.25, 95% CI 3.25-29.79), and a synchronized schedule of HIV and STI testing with PrEP visits (OR 51.85, 95% CI 19.30-139.34). No incident HIV was detected in the follow-up period. At baseline, the overall STI (CT, NG, or syphilis) prevalence was 30%, with CT at 18%, NG at 13%, and syphilis at 5%. During follow-up, the incidences were 59.08/100 person-years (py) for any STI, 33.05/100 py for CT, 29.86/100 py for NG, and 10.4/100 py for syphilis. The detection rates of CT and NG in urine samples were lower than with pharyngeal swabs and rectal swabs. The scores for convenience, confidence of correct sampling, and accuracy of self-sampling were high (7 to 8 out of 10).

**Conclusions:**

Both baseline prevalence and incidence of STI were high among MSM engaged in regular testing. A high degree of engagement in regular STI and HIV testing was positively associated with incident STI, history of health-seeking behaviors, and perceived convenience of self-sampling. Self-sampling could be introduced as a means of enhancing engagement in regular HIV and STI testing.

## Introduction

Worldwide, the prevalence of sexually transmitted infection (STI) is high in men who have sex with men (MSM), with a pooled syphilis prevalence of 7.5% (95% CI 7%-8%) [1], rectal gonorrhea prevalence of 6.1%, and rectal chlamydia prevalence of 9% [2], as summarized in systematic reviews. Although syndromic management of STI is advocated to facilitate care for infected persons, especially in limited-resource settings [3], the approach has a shortcoming of ignoring asymptomatic infections, which could be a driver of ongoing transmission [4]. In response to the high STI prevalence and high proportion of asymptomatic infections in MSM, HIV and STI testing at 3 to 6-monthly intervals has been recommended in guidelines from the US Centers for Disease Control and Prevention [5], professional bodies in other countries including Australia [6], and the Scientific Committee on AIDS and STI in Hong Kong [7]. However, adherence of MSM to regular testing, which involves undergoing HIV and STI tests at regular intervals, may not be high. In Australia, adherence of MSM to regular testing was poor, with an HIV and STI retesting rate of less than 40% at 1 year and below 20% within 6 months [8]. Similarly, the proportion of MSM undergoing regular STI testing at 6 months and 1 year was low in Europe [9-11].

Barriers such as cost, inconvenience, previous negative testing results, unawareness, confidentiality concerns, and lack of time might hinder uptake of HIV and STI tests, especially with regards to regular testing [12]. Apart from cost, the required sampling method for testing is an important factor affecting engagement in retesting [12]. Unlike urine, which is easily self-sampled, collection of pharyngeal and rectal swabs is commonly performed by health care workers in clinical settings. Self-sampled testing for HIV, *Chlamydia trachomatis* (CT), and *Neisseria gonorrhoeae* (NG) are preferred by some MSM as it overcomes the barriers of privacy concerns, time, and geographic limitations of services provided [12]. The cost of self-sampled testing for MSM could be lower by eliminating clinic attendance if the delivery cost is nominal. Combining an online service with self-sampling and self-testing may further reduce the burden of an on-site service, reserving the capacity for complex clinical management. The transition from on-site to online services for self-sampling and self-testing of STI has been shown to be effective in previous studies in the United Kingdom [13,14]. Also, a previous study of human papillomavirus (tested via oral fluid, penis, rectum specimens) in China and a study of CT and NG (tested via extragenital samples) in the United Kingdom showed comparable accuracy and consistency between self-sampled and clinician-sampled specimens in MSM [15,16].

In Hong Kong, MSM accounted for less than 20% of attendees at Social Hygiene Clinics, which are the government’s STI service. The prevalence of asymptomatic CT and NG infections in MSM attendees in 2014-2015 was 19.6% [17]. The proportion of MSM with newly diagnosed CT and NG infection has increased over time [7]. We hypothesized that engagement plays an important role in the participation of MSM in regular HIV and STI testing. In this study, we aimed to pilot-test internet-based self-sampled HIV and STI testing from multiple anatomic sites to enhance engagement of MSM in regular sexual health monitoring.

## Methods

### Participants and Study Design

In this 1-year cohort study, MSM were recruited to participate in regular testing of HIV and STIs at 3-monthly intervals. Men aged 18 years or older who had had sex with men in the past 12 months, normally lived in Hong Kong, had a negative HIV test result, and could communicate in written and spoken English or Chinese were eligible to join the study. Recruitment was performed via referral from community-based organizations, online advertisements, peers, and an HIV pre-exposure prophylaxis (PrEP) pilot study conducted by the research team. Written informed consent was obtained from each participant.

**Figure 1:**
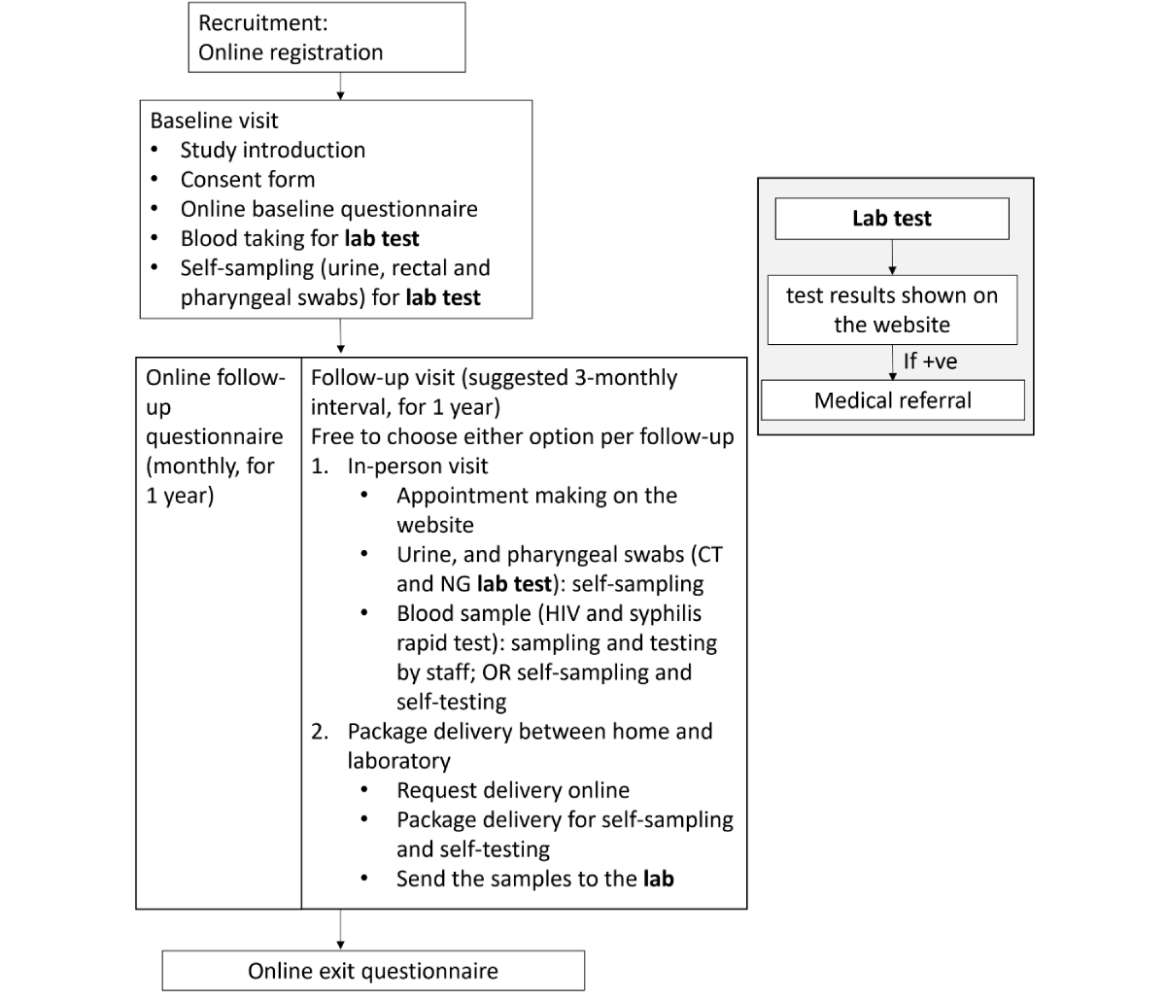
Study flowchart.

In this study, a designated website was created to describe the study procedures and provide materials about HIV and STI prevention. After registration, the same website allowed participants to complete self-administered questionnaires, make appointments for HIV and STI testing visits, and check their HIV and STI test results (Figure 1). Monthly reminders to complete questionnaires and appointment confirmation emails were sent automatically. Help desk service supported by the research team was available via WhatsApp, email, and phone calls.

With written consent at the baseline visit, participants self-collected pharyngeal swabs, rectal swabs, and urine samples for CT and NG testing by following the procedures depicted on an instruction sheet (manufacturer’s materials). Blood samples for HIV and syphilis point-of-care (POC) tests were collected by venesection. At follow-up visits, participants followed the same procedures as for the baseline visit, except that finger-prick blood samples were either self-sampled by the participants with or without assistance from research staff or venesection by a doctor. Dried blood spots (DBS) were collected using a Whatman card for HIV and syphilis laboratory testing and archiving. As an alternative to attending follow-up visits, courier delivery of kits for self-sampling for CT and NG testing and HIV and syphilis self-testing was offered. Participants were asked to upload photos of self-testing results to the system or through WhatsApp and return the self-sampled specimens for free to the laboratory through the designated courier service. Test results were inputted to the system when available so the participants could access them. Medical referral was made upon request.

Participants were asked to complete a baseline questionnaire (sociodemographic information, sexual behavioral history, PrEP use, sex networking, HIV and STI testing history, and the latest use of sexual health services), monthly follow-up questionnaires (sexual behavioral history, sex networking, HIV and STI testing history and results, scoring of the self-sampling process if participated in follow-up visits, sexual health service utilization, and PrEP use in the past 1 month), and an exit questionnaire (future plans for HIV and STI testing, preferred channels of testing, history of checking HIV and STI testing results in the system, scoring of the self-sampling process) at the end of the study. Scoring on the self-sampling process ranged between 1 (strongly disagree) and 10 (strongly agree). Chemsex engagement was defined as the use of recreational drugs before or during sex, excluding single use of either poppers or erectile dysfunction agents. Incentives via a HK $25 (US $3.20) voucher were offered to participants who had attended 3 or more visits over a 1-year period.

### Point-of-Care Testing and Laboratory Testing

The fourth generation Alere HIV Combo test was used for POC HIV testing. Participants never diagnosed with syphilis were tested with the SD BIOLINE Syphilis 3.0 (treponemal test). For participants who previously tested positive or were ever diagnosed with syphilis, their samples were further tested using the CHEMBIO DPP Syphilis Screen & Confirm (treponemal and nontreponemal test). To confirm the detection of HIV and syphilis infection, tests were performed on the collected DBS at the research laboratory. Briefly, a spot was punched from the DBS and suspended in 200 μl of phosphate-buffered saline in the well of a flat-bottomed uncoated microtiter plate. The plate was incubated at 4° C for 2 hours, and 150 μl of the eluate was subsequently used as the specimen for testing. Eluted DBS was separately tested for HIV and syphilis antibody using a commercial ELISA kit (Murex HIV AG/Ab Combo and Murex ICE-Syphilis, DiaSorin, Saluggia VC, Italy, respectively) following the manufacturers’ instructions. CT and NG nucleic acid amplification tests were performed on 3 types of specimens, namely, urine, rectal swabs, and pharyngeal swabs. CT and NG were detected using the Aptima Combo 2 Assay (Hologic, Marlborough, MA).

### Ethical Approval

Ethical approval from the Joint Chinese University of Hong Kong-New Territories East Cluster Clinical Research Ethics Committee was obtained (approval number: CREC2019.167).

### Data Analysis

MSM participants who had made at least 3 visits spanning over at least 180 days were regarded as fully engaged, while the rest were classified as partially engaged. Baseline characteristics and participation experiences during the follow-up period were compared between the 2 engagement groups in bivariable logistic regression models. Scores for the self-sampling processes were summarized, and their changes (dichotomized: 8-10 points for convenience, confidence, and accuracy coded as 1; 3-10 points for discomfort coded as 1) by visit were examined in a binary logistic generalized estimating equation (GEE), following unstructured working correlation matrices. Sensitivity analyses were performed with different cut-off values for the dependent variables in the GEE models.

We estimated the baseline STI prevalence by dividing the number of MSM with a positive STI result (including any sites for CT, any sites for NG, or reactive syphilis or HIV antibody) by the total number of MSM with baseline STI test results. The 95% CI was estimated using a binomial exact test. Incident STI referred to the first positive STI result after a negative result. Time to event was the interval (in months) from the baseline visit to either the date of an incident STI or the last visit, whichever was earlier. Incidence and 95% CI were calculated based on the Poisson distribution assumption. All statistical analyses were performed in SPSS 25 (IBM Corp, Armonk, NY), and complete case analyses were performed.

## Results

### Characteristics of Participants at Baseline

Between August 10, 2019, and October 9, 2020, a total of 242 participants were registered in the study; 206 MSM had provided consent and attended the baseline visit. Per the eligibility criteria, the 2 MSM who tested HIV-positive at baseline (2/206, 1%; 95% CI 0%-2%) were excluded from the study. Among the 204 successfully recruited MSM, the median age was 31 (IQR 26-39) years, 92.7% (189/204) were local Chinese, 92.5% (185/200) had attained a secondary educational level or higher, and 80.6% (162/201) were employed (Table 1). Most participants (158/204, 77.5%) had a synchronized schedule for HIV and STI testing with the PrEP study visit (ie, co-enrollment in the PrEP study). At baseline, 38.0% (76/200) reported a history of chemsex engagement, 87.0% (174/200) reported a history of group sex, 36.4% (68/187) reported a history of an STI diagnosis, 58.0% (116/200) had experience with PrEP, and 19.6% (39/199) had previously taken post-exposure prophylaxis (PEP) against HIV.

At baseline, the overall STI prevalence was 30% (95% CI 24%-36%), with CT at 18% (95% CI 12%-23%), NG at 13% (95% CI 8%-17%), and syphilis at 5% (95% CI 2%-8%). By anatomic site, the highest proportion of samples with a positive result was rectal swabs, at 12.9% (26/201) for CT, and pharyngeal swabs, at 9.0% (18/199) for NG. The lowest proportion of samples testing positive was in urine samples, at only 3.9% (8/202) for CT and 1.5% (3/201) for NG.

**Table 1:** Characteristics of recruited men who have sex with men (n=204).

Variables				Results
**Sociodemographic variables**				
	Age (years), median (IQR) (n=203)		31 (26-39)	
	**Recruitment source (n=200), n (%)**			
		NGOs^a^	69 (34.5)	
		Other research projects	109 (54.5)	
		Online	10 (5.0)	
		Peers	12 (6.0)	
	**Local Chinese (n=204), n (%)**			
		No	15 (7.4)	
		Yes	189 (92.6)	
	**Education level (n=200), n (%)**			
		Secondary or below	15 (7.5)	
		Postsecondary	185 (92.5)	
	**Employed (n=200), n (%)**			
		No	38 (19.0)	
		Yes	162 (81.0)	
	**Monthly income level (HK $^b^; n=200), n (%)**			
		0	17 (8.5)	
		1-15,000	40 (20.0)	
		15,001-30,000	72 (36.0)	
		30,001-50,000	42 (21.0)	
		>50,000	29 (14.5)	
**Channels ever used for seeking sex partners (n=200), n (%)**				
	Local physical venue		142 (71.0)	
	Overseas physical venue		79 (39.5)	
	Virtual venue		186 (93.0)	
**History of sexual behavior (n=200)**				
	**Group sex, n (%)**			
		Never	26 (13.0)	
		Ever	174 (87.0)	
	**Chemsex engagement, n (%)**			
		Never	124 (62.0)	
		Ever	76 (38.0)	
	**Sex with a woman, n (%)**			
		Never	170 (85.0)	
		Ever	30 (15.0)	
**STI^c^ diagnosis (n=187), n (%)**				
	Never		119 (63.6)	
	Ever		68 (36.4)	
**HIV prevention measures**				
	**Knowledge and usage of PrEP^d^ (n=200), n (%)**			
		Never heard of	2 (1.0)	
		Have heard of but never taken	82 (41.0)	
		Have taken	116 (58.0)	
	**Knowledge and usage of PEP^e^ (n=198), n (%)**			
		Never heard of	3 (1.5)	
		No	156 (78.8)	
		Yes	39 (19.7)	

^a^NGOs: nongovernmental organizations.

^b^US $1=HK $7.8.

^c^STI: sexually transmitted infection.

^d^PrEP: pre-exposure prophylaxis.

^e^PEP: post exposure prophylaxis.

### Self-Sampling Preference for Regular STI Testing at Baseline

From the baseline questionnaire (n=200), 65.5% (131/200) of MSM had heard of self-sampling for STI or HIV testing, and 82.5% (165/200) indicated a willingness to try (Table 2). Most MSM preferred POC testing with samples collected by a trained staff member (165/200, 82.5%), followed by self-testing (103/200, 51.5%) and sampling by health care workers with consequent laboratory testing (64/200, 32.0%). The lowest proportion preferred self-sampling with consequent laboratory testing (42/200, 21.0%). Most MSM supported regular HIV testing (192/194, 99.0%) and STI testing (187/194, 96.4%) at least once every 2 years, with more than one-half expecting to undergo testing once every 3 months (149/194, 76.8% for HIV testing; 129/194, 66.5% for STI testing). Among all sampling methods, finger prick, saliva, and urine were well-accepted (>70% MSM replied each was totally acceptable) by the participants, while urethral sampling was the least acceptable method (58/189, 30.7% of MSM replied it was totally acceptable).

**Table 2:** Preference of HIV and sexually transmitted infection (STI) testing at baseline (n=200).

Preferences				Results, n (%)
**Testing frequency (n=194)**				
	**Appropriate HIV testing frequency**			
		Test whenever necessary	2 (1.0)	
		Once every 2 years	1 (0.5)	
		Once per year	3 (1.5)	
		Once every 6 months	24 (12.4)	
		Once every 3 months	149 (76.8)	
		Once per month	15 (7.7)	
	**Appropriate STI testing frequency**			
		Test whenever necessary	7 (3.6)	
		Once every 2 years	2 (1.0)	
		Once per year	10 (5.2)	
		Once every 6 months	34 (17.5)	
		Once every 3 months	129 (66.5)	
		Once per month	12 (61.9)	
**Heard of self-sampled HIV and STI test (n=200)**				
	No		69 (34.5)	
	Yes		131 (65.5)	
**Willing to try self-sampling for HIV and STI testing** **(n=200)**				
	Yes, even if paid		28 (14.0)	
	Yes, if free		137 (68.5)	
	Not decided yet		21 (10.5)	
	No		14 (7.0)	
**Type of sampling and testing preferred (n=200)**				
	Self-test		103 (51.5)	
	Point-of-care test with samples collected by a trained staff member		165 (82.5)	
	Self-sampling and tested by a lab		42 (21.0)	
	Sampled by an HCW^a^ and tested by a lab		64 (32.0)	
**Level of acceptability of the different sampling methods**				
	**Giving blood (n=198)**			
		Totally acceptable	116 (58.6)	
		Acceptable	73 (36.9)	
		Unacceptable	4 (2.0)	
		Totally unacceptable	5 (2.5)	
	**Finger prick (n=197)**			
		Totally acceptable	147 (74.6)	
		Acceptable	44 (22.3)	
		Unacceptable	1 (0.5)	
		Totally unacceptable	5 (2.5)	
	**Urine sample (n=199)**			
		Totally acceptable	153 (76.9)	
		Acceptable	42 (21.1)	
		Unacceptable	0 (0)	
		Totally unacceptable	4 (2.0)	
	**Saliva sample (n=199)**			
		Totally acceptable	155 (77.9)	
		Acceptable	40 (20.1)	
		Unacceptable	0 (0)	
		Totally unacceptable	4 (2.0)	
	**Rectal swab (n=193)**			
		Totally acceptable	94 (48.7)	
		Acceptable	87 (45.1)	
		Unacceptable	8 (4.2)	
		Totally unacceptable	4 (2.1)	
	**Pharyngeal swab (n=190)**			
		Totally acceptable	99 (52.1)	
		Acceptable	76 (40.0)	
		Unacceptable	11 (5.8)	
		Totally unacceptable	4 (2.1)	
	**Urethral swab (n=189)**			
		Totally acceptable	58 (30.7)	
		Acceptable	88 (46.6)	
		Unacceptable	32 (16.9)	
		Totally unacceptable	11 (5.8)	

^a^HCW: health care worker.

### Comparison of Participants by Study Engagement Level

Regarding study visits, among 204 participants, 28 (13.7%) attended the baseline visit only, while 29 (14.2%) visited twice, and 147 (72.1%) attended 3 or more visits including 1 visit with a follow-up period shorter than 180 days (Multimedia Appendix 1). Baseline characteristics were not significantly different between MSM with full (at least 3 visits) and partial (less than 3 visits) engagement (Table 3). MSM with an incident STI in the follow-up period (odds ratio [OR] 4.23, 95% CI 1.63-10.94), who had ever sought a medical referral following STI detection during the study (OR 10.25, 95% CI 3.25-29.79), and who had a synchronized schedule of HIV and STI testing with the PrEP visit (OR 51.85, 95% CI 19.30-139.34) were more likely to be fully engaged than partially engaged in the study.

**Table 3:** Comparison of participants’ baseline characteristics by study engagement level.

Characteristics				Partial engagement (<3 visits; n=58), n (%)		Full engagement (≥3 visits; n=146), n (%)		Bivariable logistic regression analysis				
								OR^a^ (95% CI)		*P* value		
**Sociodemographic variables**												.76
	Age (years)^b,c^			30 (25-37)		31 (27-39)		1.01 (0.97-1.04)				
	**Local Chinese^c^**										.87	
		No	4 (6.9)		11 (7.5)		Reference					
		Yes	54 (93.1)		135 (92.5)		0.91 (0.28-2.98)					
	**Education level^d^**										.98	
		Secondary or below	4 (7.4)		11 (7.5)		Reference					
		Postsecondary	50 (92.6)		135 (92.5)		0.98 (0.30-3.23)					
	**Employed^d^**										.27	
		No	13 (24.1)		25 (17.1)		Reference					
		Yes	41 (75.9)		121 (82.9)		1.53 (0.72-3.27)					
	**Monthly income level (HKD)^d,e^**											
		0	5 (9.3)		12 (8.2)		Reference		—^f^			
		1-15,000	16 (29.6)		24 (16.4)		0.63 (0.18-2.12)		.45			
		15,001-30,000	14 (25.9)		58 (39.7)		1.73 (0.52-5.70)		.37			
		30,001-50,000	10 (18.5)		32 (21.9)		1.33 (0.38-4.71)		.66			
		>50,000	9 (16.7)		20 (13.7)		0.93 (0.25-3.42)		.91			
**Channels ever used for seeking sex partners^d^**												
	Local physical venue			35 (64.8)		107 (73.3)		1.49 (0.76-2.91)		.24		
	Overseas physical venue			19 (35.2)		60 (41.1)		1.29 (0.67-2.46)		.45		
	Virtual venue			51 (94.4)		135 (92.5)		0.72 (0.19-2.69)		.63		
**History of sexual behavior**												
	**History of group sex^d^**										.06	
		Never	11 (20.4)		15 (10.3)		Reference					
		Ever	43 (79.6)		131 (89.7)		2.23 (0.95-5.23)					
	**History of chemsex engagement^d^**										.42	
		Never	31 (57.4)		93 (63.7)		Reference					
		Ever	23 (42.6)		53 (36.3)		0.77 (0.41-1.45)					
	**History of STI^g^ diagnosis^h^**										.45	
		Never	34 (68.0)		85 (62.0)		Reference					
		Ever	16 (32.0)		52 (38.0)		1.30 (0.65-2.58)					
**HIV and STI testing**												
	**Acceptance of regular HIV testing^i^**										—	
		No	2 (3.8)		0 (0)		—					
		Yes	51 (96.2)		141 (100)		—					
	**Acceptance of regular STI testing^i^**										.36	
		No	3 (5.7)		4 (2.8)		Reference					
		Yes	50 (94.3)		137 (97.2)		2.06 (0.44-9.51)					
	**Types of sampling and testing preferred^d^**											
		Self-test	28 (51.9)		75 (51.4)		0.98 (0.53-1.83)		.95			
		Point-of-care test with samples collected by a trained staff member	49 (90.7)		116 (79.5)		0.39 (0.14-1.08)		.07			
		Self-sampling and testing by a lab	10 (18.5)		32 (21.9)		1.24 (0.56-2.72)		.60			
		Sample taken by an HCW^j^ and tested by a lab	11 (20.4)		53 (36.3)		2.23 (1.06-4.68)		.03			
**Research outcomes and participation**												
	**Baseline positive STI result^c^**										.37	
		No	38 (65.5)		105 (71.9)		Reference					
		Yes	20 (34.5)		41 (28.1)		0.74 (0.39-1.42)					
	**Incident STI during the follow-up^k^**										.003	
		No	24 (80.0)		71 (48.6)		Reference					
		Yes	6 (20.0)		75 (51.4)		4.23 (1.63-10.94)					
	**Sought medical referral during the study^c^**										<.001	
		No	54 (93.1)		83 (56.8)		Reference					
		Yes	4 (6.9)		63 (43.2)		10.25 (3.52-29.79)					
	**Synchronized schedule with PrEP^l^ study visit^c^**										<.001	
		No	40 (69.0)		6 (4.1)		Reference					
		Yes	18 (31.0)		140 (95.9)		51.85 (19.30-139.34)					

^a^OR: odds ratio.

^b^Median (IQR).

^c^n=204.

^d^n=200.

^e^US $1=HK $7.8.

^f^Not applicable.

^g^STI: sexually transmitted infection.

^h^n=187.

^i^n=194.

^j^HCW: health care workers.

^k^n=176.

^l^PrEP: pre-exposure prophylaxis.

### Sampling and Testing During Follow-up

During the follow-up periods, of 762 sets of samples collected, 638 sets (83.7%) were blood sampled by staff for HIV and syphilis rapid tests with self-sampling of swabs and urine, 44 sets (5.8%) were self-sampled (DBS, swabs, urine samples) and self-tested for HIV and syphilis at the site, and 80 sets (10.5%) were self-sampled (DBS, swabs, urine samples) and self-tested for syphilis at home with delivery to the laboratory. From visits 2 to 5, there was a decreasing proportion of participants who selected the home delivery option and the self-sampling with self-testing at the site option (Table 4).

Regarding the scoring after self-sampling, the median score was 8 (IQR 5-9) for convenience, 7 (IQR 4-9) for confidence of correct self-sampling, 7 (IQR 5-9) for accurate reflection of his HIV or STI status from self-sampled specimens, and 3 (IQR 2-5) for discomfort during self-sampling at the baseline visit (Table 5). The proportions of high scores (8-10 points in Models A, B, and C; 3-10 points in Model D) were similar between baseline and follow-up visits 2 to 5, without significant changes identified in the GEE models. In the sensitivity analyses, cut-off values of 7-10 and 9-10 for Models A, B, and C and values of 2-10 and 4-10 for Model D showed the same conclusion. In reference to the median scores for visits 1 to 5, 55.3% (83/150) of MSM scored 8 to 10 for convenience, 45.1% (69/153) scored 8 to 10 for confidence in correct sampling, 53.2% (82/154) scored 8 to 10 for accuracy, and 39.3% (59/150) scored 1 to 2 for discomfort. A high median score for convenience was positively associated with full study engagement (OR 2.68, 95% CI 1.14-6.30) in the logistic regression model, but no other markers had significant associations.

There was no incident HIV infection in the follow-up period. Among MSM with at least 2 rounds of testing performed during a median follow-up period of 12.53 (IQR 9.62-14.71) months, the incidence of any STI was 59.1 per 100 person-years (py; 81/137.11 years, 95% CI 47.2-73.1 per 100 py), with the highest incidence for CT (51/154.29 years; 33.1, 95% CI 24.9-43.1 per 100 py), followed by NG (48/160.77 years; 29.9, 95% CI 22.3-39.3 per 100 py) and syphilis (18/173.08 years; 10.4, 95% CI 6.4-16.1 per 100 py). By anatomic site, the highest proportion of samples testing CT-positive was in rectal swabs (46/539, 8.5%). The highest proportion of samples testing NG-positive was in pharyngeal swabs (37/546, 6.8%). Among urine samples, only 2.5% (14/554) were CT-positive, and 1.6% (9/555) were NG-positive. Comparing the positivity of one or more STI conditions at each or all anatomic sites during the follow-up in bivariable logistic regression analysis, no difference in STI positivity was identified between sampling by staff or participants nor between in-person visits or home delivery. However, no samples tested positive for syphilis in the home delivery group and self-sampling at site group, with the same syphilis rapid test kit (treponemal and nontreponemal test) provided to participants with or without a past history of syphilis.

A total of 113 participants completed the exit questionnaire. They were more likely to be fully engaged (OR 11.50, 95% CI 5.33-24.80) and be co-enrolled in the PrEP study (OR 5.02, 95% CI 2.41-10.46), compared with those who did not complete the exit questionnaire. Their sociodemographic characteristics (locality, employment status, and educational level) were however not significantly different. In the exit questionnaire data from the 113 participants, 103 (91.2%) had checked the testing results from the designated website, and 100 (99%) replied having a plan for regular STI testing (at least once a year) after the study.

**Table 4:** Distribution of sampling and testing options by participants and staff over visits (n=762).

Visit number	Number of participants who completed the visit	In-person visit, n (%)		Home delivery, n (%)
		Blood sampled by staff, rapid test for HIV and syphilis by staff (n=638)	Blood self-sampled and self-tested for HIV and syphilis at the site (n=44)	Blood self-sampled and self-tested for syphilis at home (n=80)
1	204	204 (100)	0 (0)	0 (0)
2	176	114 (64.8)	29 (16.5)	33 (18.8)
3	147	111 (75.5)	9 (6.1)	27 (18.4)
4	124	107 (86.3)	3 (2.4)	14 (11.3)
5	79	76 (96.2)	1 (1.3)	2 (2.5)
6	20	15 (75.0)	2 (10.0)	3 (15.0)
7	8	7 (87.5)	0 (0)	1 (12.5)
8	3	3 (100)	0 (0)	0 (0)
9	1	1 (100)	0 (0)	0 (0)

**Table 5:** Post-self-sampling scores (ranging between 1 [strongly disagree] and 10 [strongly agree]) and their changes across visits in binary logistic generalized estimating equation (GEE) models.

Model and visit number		Participants, n			Median (IQR)		High score^a^, n (%)		GEE model results		
									Exp(B)		*P* value
**Model A: It's convenient**											
	1		60	8 (5-9)		31 (52)		1		—^b^	
	2		105	8 (4-10)		57 (54)		1.03		.91	
	3		97	8 (5-9)		52 (54)		0.93		.81	
	4		75	8 (6-10)		45 (60)		1.11		.75	
	5		51	8 (5-9)		31 (61)		1.45		.28	
	Intercept		—	—		—		1.19		.48	
**Model B: I am confident to self-sample correctly**											
	1		62	7 (4-9)		23 (37)		1		—	
	2		106	8 (5-9)		55 (52)		1.33		.34	
	3		97	7 (5-9)		46 (47)		1.05		.87	
	4		76	8 (5-10)		42 (55)		1.46		.24	
	5		50	8 (5-9)		26 (52)		1.71		.11	
	Intercept		—	—		—		0.77		.30	
**Model C: I am confident that self-sampled specimens can accurately reflect my HIV or STI status**											
	1		61	7 (5-9)		30 (49)		1		—	
	2		108	8 (5-9)		61 (58)		0.81		.61	
	3		96	7 (5-9)		47 (49)		0.67		.29	
	4		76	8 (5-10)		42 (55)		0.78		.51	
	5		49	8 (6-9)		29 (58)		0.70		.47	
	Intercept		—	—		—		1.52		.22	
**Model D: I feel discomfort during self-sampling**											
	1		57	3 (2-5)		33 (58)		1		—	
	2		101	3 (2-6)		69 (68)		1.95		.10	
	3		93	3 (2-6)		57 (61)		1.42		.38	
	4		75	3 (2-5)		39 (52)		1.11		.81	
	5		49	4 (2-7)		32 (65)		0.93		.82	
	Intercept		—	—		—		1.08		.82	

^a^The high score was coded 1 as a binary dependent variable in the GEE models: scores ≥8 for models A, B, and C and ≥3 for model D.

^b^Not applicable.

## Discussion

### Principal Findings

This is a longitudinal study involving a designated website and self-sampling method to enhance regular HIV and STI testing in MSM. Despite the low proportion (21.0%) of participants indicating a preference for self-sampled testing at baseline, the overall completion rate of regular self-sampled testing was high, at 72.1%. Scoring of the self-sampling process by participating MSM was high for its convenience, confidence of correct sampling, and perceived accurate reflection of STI status, and only very mild discomfort was reported.

### Comparison With Prior Work

There was a positive association between seeking a medical referral subsequent to the detection of an incident STI and full engagement in regular HIV and STI testing. The detection of new STI may have alerted MSM about their health risk regardless of experiencing symptoms. With treatment as a follow-up action, STI knowledge may be enhanced in this group. The higher STI knowledge may have promoted engagement in frequent STI testing [18]. On the other hand, the compliance rate for MSM with a synchronized schedule of HIV and STI testing with a PrEP visit was higher than regular HIV and STI testing as an isolated component. Among MSM on PrEP for HIV prevention, a recent HIV test result is often a precondition for a PrEP refill [19]. Bundling STI testing with HIV testing for a PrEP refill, as has been packaged in many PrEP implementation studies [20-22], could be a useful strategy for individuals at higher risk of HIV and STI. Self-sampled testing may further increase uptake by providing the flexibility to undergo testing.

MSM who scored the convenience of self-sampling higher in the first 5 visits were more likely to be fully engaged with regular testing during participation in the study. This is consistent with the higher acceptability of internet-based testing with self-sampling, an approach that saved travel and clinic waiting times, as concluded in a systematic review [23]. Our participants’ preferences for self-sampling and self-testing at baseline were not significantly different by socioeconomic factors including educational level, Chinese locality, and employment. Potential barriers for self-sampling include a lack of confidence in correct self-sampling, inaccurate results in the self-sampled samples, and discomfort [23]. Although these factors were not significantly associated with full engagement, more than one-half of MSM scored confidence and accuracy high, and around 40% provided low scores for discomfort.

In this MSM cohort, the prevalence of overall STI was high at baseline, at 31%, which was higher than the baseline STI prevalence in PrEP studies in Amsterdam (17%) [20] and Australia (13.7%) [24], and the baseline CT and NG prevalences in this study (26%) were also higher than in a previous local study of CT and NG with MSM attending an STI clinic (19.6%) [17]. The incidence of any STI in the follow-up period of this study was however lower than that in previous PrEP studies [21,22]. The proportion of positive test results in extragenital site samples was higher than that in urine samples. The rate of detection of CT and NG from multisite samples highlights the insufficiency of collecting urine samples alone for CT and NG testing in MSM. This observation was consistent with previous studies [21,22] and supported by guidelines for CT and NG screening at extragenital sites in MSM [5-7] and results from a cost-effectiveness analysis [25].

### Limitations

There were a few limitations in this study. First, a good proportion (77%) of participants had concurrently joined the PrEP study being conducted by the research team, which allowed synchronized scheduling of appointments for the 2 studies. The completion rate of the regular testing study was likely to be affected partly by the PrEP study. Comparison of PrEP-naïve and experienced participants was not performed because of the unbalanced distribution. Second, CT and NG test results from self-collected samples may be affected by the sampling technique of the participants. However, with high positivity rates and high proportion of detectable CT or NG in the test kits, incorrect sampling was unlikely to be a major concern. Studies have shown comparable accuracy and consistency results between self-sampled and clinician-sampled specimens [15,16]. Third, we did not use a validated tool to score self-sampling. As convenience was identified as a significant factor associated with the degree of engagement, further development of an evaluation tool for self-sampling may be useful. Fourth, self-selection bias might have arisen, with MSM at higher risk of HIV or STI being attracted to participate in the study, and recall bias might exist in the questionnaire survey even though it was completed on a monthly basis. Fifth, there was no control group who had samples taken by health care workers nor a non-web-based booking system, so we were unable to directly assess the outcome of the program.

### Conclusion

The STI burden for MSM in the community in Hong Kong was high. MSM with an incident STI, seeking medical referrals, and who scored the convenience of self-sampling high were more likely to become fully engaged in a regular testing program. Self-sampling and the functioning of a website could enhance regular HIV and STI testing, supplementing conventional methods for implementing testing.

## Appendix

Multimedia Appendix 1 Trends of retention in the study.

## Abbreviations

CT: Chlamydia trachomatis
DBS: dried blood spots
GEE: generalized estimating equation
MSM: men who have sex with men
NG: Neisseria gonorrhoeae
OR: odds ratio
PEP: post-exposure prophylaxis
POC: point-of-care
PrEP: pre-exposure prophylaxis
py: person-years
STI: sexually transmitted infection
