# Supplementary figures and images for: Regular Testing of HIV and Sexually Transmitted Infections With Self-Collected Samples From Multiple Anatomic Sites to Monitor Sexual Health in Men Who Have Sex With Men: Longitudinal Study
Source: JMIR Form Res. 2022 Nov 18;6(11):e40996. doi: 10.2196/40996 (PMC9719057; doi:10.2196/40996)

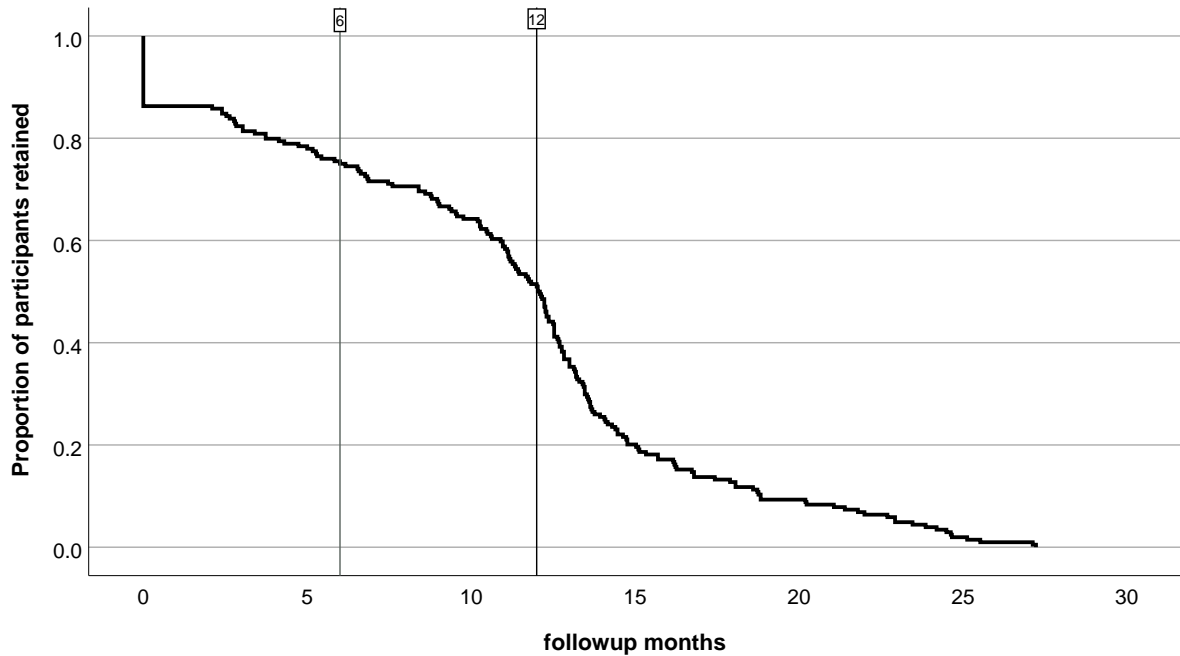

Supplement: Multimedia Appendix 1 [file formative_v6i11e40996_app1.pdf]
